# VTA BDNF enhances social stress-induced compulsive cocaine bingeing

**DOI:** 10.18632/oncotarget.13995

**Published:** 2016-12-16

**Authors:** Junshi Wang, Ryan M. Bastle, Ella M. Nikulina

**Affiliations:** ^1^ Department of Basic Medical Sciences, College of Medicine, The University of Arizona, Phoenix, AZ, USA

**Keywords:** BDNF, ΔFosB, ventral tegmental area, self-administration, cocaine, Neuroscience

Every day in the United States, 120 people die as a result of drug overdose, a rate that has more than doubled from 1999 to 2013 [1]. The degree of vulnerability to addiction is determined by the interaction between genetic and environmental factors. Social stress is an environmental factor some experience on a daily basis (e.g., domestic abuse, bullying, work-related issues, etc.). Since people under social stress are more vulnerable to become addictive to drugs of abuse [2], it is critical to understand the brain mechanisms underlying such vulnerability, thereby providing potential therapeutic targets. The rodent social defeat model has been used to mimic social stress in humans and to examine social stress-induced brain changes. In this procedure, a dominant rat psychologically threatens and physically attacks a subordinate rat intermittently over the course of 10 days. Defeated rats exhibit enhanced drug-taking behavior, as well as have increased expression of addiction-related molecules in critical reward-related brain regions [3, 4]. More specifically, defeated rats have increased expression of the transcription factor ΔFosB in the nucleus accumbens (NAc) and increased brain-derived neurotrophic factor (BDNF) expression in the ventral tegmental area (VTA) [3, 4]. The VTA is enriched with dopamine neurons that are activated in response to drugs of abuse and project to the NAc to signal attention and motivation towards rewarding stimuli. BDNF promotes neural growth and plasticity in response to environmental stimuli, whereas ΔFosB in the NAc regulates sensitivity to cocaine and susceptibility to stress [5]. Furthermore, increasing VTA BDNF expression enhances NAc ΔFosB levels [6]. Therefore, we [7] investigated whether increased VTA BDNF also enhances social stress-induced vulnerability to addiction.

To test this hypothesis, VTA BDNF expression was artificially increased with an adeno-associated viral vector (AAV), where control groups only received an AAV carrying the gene for green fluorescent protein (GFP). One week later, rats were exposed to either intermittent social defeat stress or were briefly handled. To measure drugtaking behavior, rats were trained to lever press for cocaine infusions (i.e., self-administration). Initial acquisition and maintenance of cocaine self-administration was measured in daily 2-h sessions. Following maintenance, motivation for cocaine was measured by placing the rats on a high effort progressive ratio (PR) schedule of reinforcement, where the number of lever presses the animal needs to acquire a single infusion increases exponentially (often in the hundreds by the end of the session). Following PR testing, compulsive cocaine intake was measured by allowing rats to self-administer cocaine during a single 12-h session (i.e., binge). While no group differences were observed during initial acquisition and maintenance of cocaine self-administration, stressed groups exhibited increased motivation for cocaine on the PR schedule compared to the non-stressed groups, regardless of virus. Interestingly, socially defeated animals with intra-VTA BDNF overexpression exhibited the highest amount of cocaine intake during the 12-h binge session, whereas non-defeated animals with intra-VTA BDNF overexpression did not increase binge-like intake compared to controls. These findings suggest that VTA BDNF cannot induce vulnerability to drugs of abuse by itself, but facilitates the effects of stress on compulsive cocaine-taking behavior.

Following behavioral testing, BDNF in the VTA and ΔFosB in the NAc were quantified using immunohistochemistry. Similar to the binge findings, socially defeated rats that received intra-VTA BDNF overexpression had the highest level of VTA BDNF among all groups. Furthermore, a significant positive correlation between VTA BDNF and cocaine intake during the 12-h binge was observed. Consistent with previous findings, VTA BDNF overexpression enhanced levels of ΔFosB in the NAc. Notably, this effect persisted even after chronic cocaine self-administration. Given that the VTA is a primary source of BDNF that is released in the NAc, increases in ΔFosB may occur through enhanced VTA BDNF-stimulated signaling that alters gene expression in the NAc. This potential transcriptional mechanism may underlie BDNF-induced neuroplasticity in the brain reward circuitry, and may be the critical link between stress and escalated responsivity to drugs of abuse. Overall, these behavioral and neurochemical results suggest that the synergy between VTA BDNF and social stress leads to greater binge-like cocaine self-administration. In human addicts, cocaine use typically occurs in binges, and this type of drug-taking behavior is prone to result in overdose. Therefore, this study identified VTA BDNF as a risk factor for stress-induced binge-like cocaine intake and could provide a novel target for preventative therapeutics.
